# Induction of NK cell reactivity against acute myeloid leukemia by Fc-optimized CD276 (B7-H3) antibody

**DOI:** 10.1038/s41408-024-01050-6

**Published:** 2024-04-18

**Authors:** Sylwia A. Stefańczyk, Ilona Hagelstein, Martina S. Lutz, Stefanie Müller, Samuel J. Holzmayer, Grace Jarjour, Latifa Zekri, Jonas S. Heitmann, Helmut R. Salih, Melanie Märklin

**Affiliations:** 1 Clinical Collaboration Unit Translational Immunology, German Cancer Consortium (DKTK), Department of Internal Medicine, University Hospital of Tübingen, Tübingen, Germany; 2 Cluster of Excellence iFIT (EXC 2180) ‘Image-Guided and Functionally Instructed Tumor Therapies’, Eberhard Karls University of Tübingen, Tübingen, Germany

**Keywords:** Cancer immunotherapy, Leukaemia

## Abstract

Acute myeloid leukemia (AML) remains a therapeutic challenge despite recent therapeutic advances. Although monoclonal antibodies (mAbs) engaging natural killer (NK) cells via antibody-dependent cellular cytotoxicity (ADCC) hold promise in cancer therapy, almost none have received clinical approval for AML, so far. Recently, CD276 (B7-H3) has emerged as a promising target for AML immunotherapy, due to its high expression on leukemic blasts of AML patients. Here, we present the preclinical development of the Fc-optimized CD276 mAb 8H8_SDIE with enhanced CD16 affinity. We demonstrate that 8H8_SDIE specifically binds to CD276 on AML cell lines and primary AML cells and induces pronounced NK cell activation and degranulation as measured by CD69, CD25, and CD107a. Secretion of IFNγ, TNF, granzyme B, granulysin, and perforin, which mediate NK cell effector functions, was induced by 8H8_SDIE. A pronounced target cell-restricted lysis of AML cell lines and primary AML cells was observed in cytotoxicity assays using 8H8_SDIE. Finally, xenograft models with 8H8_SDIE did not cause off-target immune activation and effectively inhibited leukemia growth in vivo. We here present a novel attractive immunotherapeutic compound that potently induces anti-leukemic NK cell reactivity in vitro and in vivo as treatment option for AML.

## Background

Acute myeloid leukemia (AML) is a heterogeneous aggressive disease characterized by clonal expansion of myeloid progenitor cells with reduced ability to differentiate [1]. AML is the most common form of acute leukemia in adults, typically diagnosed at a median age of 68 years [2]. The majority of AML patients have a poor prognosis and a high risk of relapse [3]. In the past, numerous immunotherapy strategies have been preclinically evaluated for the treatment of AML, such as mono- or bispecific antibodies targeting various tumor-associated antigens [4–7]. Although many clinical trials are currently evaluating immunotherapeutic approaches such as the immune checkpoint inhibitor PD-1 Nivolumab (NCT03825367), a chimeric antigen receptor (CAR) T cells specific for CD33 (NTC04835519) or a bispecific FLT3xCD3 antibody (NCT05143996), the standard of care for most AML patients remains chemotherapy regimens or allogeneic stem cell transplantation [8–10]. Recently, several new treatments have received FDA approval for the treatment of AML, including the FLT-3 inhibitors Midostaurin and Quizartinib. However, it’s important to note that achieving complete remission and ultimately cure for AML still remains a challenge for many patients. This highlights the urgent need for new therapeutic targets and approaches [11].

The introduction of monoclonal antibodies (mAbs), such as the anti-human epidermal growth factor receptor 2 (HER2)/neu mAb Trastuzumab and the CD20 mAb Rituximab, has improved treatment options for patients with HER2-positive breast cancer and B cell malignancies, respectively [12, 13]. While the introduction of therapeutic antibodies has revolutionized cancer treatment, there is still much room for improvement. Many patients either do not respond at all or experience only short-lived benefits. In addition, there is a significant shortage of approved therapeutic antibodies for several cancers, particularly AML. To overcome these challenges, strategies are being explored to enhance the immunostimulatory properties of mAbs, for example by increasing their ability to induce antibody-dependent cellular cytotoxicity (ADCC). An example of an ADCC-optimized mAb targeting FLT3 in the minimal residual disease state of AML has shown promising results in phase I clinical trials (NCT02789254) [14].

In hematological cancers, ADCC is one of the most important effector mechanisms induced by mAb treatment, with natural killer (NK) cells acting as the primary effector cell population [15, 16]. To enhance ADCC, the Fc region of a mAb can be modified either by optimizing glycosylation patterns, as seen with the FDA-approved glycol-optimized CD20 mAb obinutuzumab for the treatment of B cell malignancies, or by introducing amino acid substitutions (e.g., S239D/I332E, SDIE) [17]. Such optimization strategies aim to increase the affinity of the Fc part to the Fcγ receptor (FcγR) in general, with a stronger effect on the activating FcγRIIIa/CD16a compared to the inhibitory FcγRIIb/CD32b [18]. In order to implement mAbs as a successful treatment for additional cancer entities, it is also critical to identify novel target antigens that are widely expressed on tumor cells, while ideally absent on healthy cells. Several promising targets have been analyzed in the past, some of which are also associated with prognosis in AML [19–23].

The B7 homolog 3 (CD276 or B7-H3), a member of the B7 immune checkpoint family is rather specifically expressed in many human cancers, including glioma, ovarian cancer, neuroblastoma, lung adenocarcinoma, pancreatic cancer, certain sarcomas, and AML. Overexpression of CD276 is associated with an unfavorable disease course and poor patient prognosis, possibly due to inhibition of T cell and NK cell reactivity [24–26]. Accordingly, CD276 has emerged as promising target antigen for many therapeutic approaches, that are currently under investigation.

Here, we characterized the expression of CD276 in AML cell lines and patient cells, and validated a novel Fc-optimized (SDIE modification) CD276 mAb termed 8H8_SDIE for the treatment of AML.

## Materials and methods

### Patient samples

Peripheral blood mononuclear cells (PBMC) were collected from the blood of AML patients (*n* = 68) at primary diagnosis and from healthy volunteers (*n* = 13) of different ages and sexes. Written informed consent was obtained in accordance with the Declaration of Helsinki. PBMC were isolated by ficoll density gradient centrifugation (Thermo Fisher Scientific, Waltham, MA, USA), kept viable in liquid nitrogen and then randomly selected for each experiment. Cryopreserved PBMC were cultured in RPMI1640 media, GlutaMAX Supplement (Thermo Fisher Scientific) supplemented with 10% heat-inactivated fetal calf serum (PAN-biotech, Aidenbach, Germany), 100 U/ml penicillin (Sigma-Aldrich, St. Louis, USA) and 100 μg/ml streptomycin (Sigma-Aldrich), at 37 °C with 5% CO_2_.

### Cell lines

The AML cell lines (EOL-1, HL-60, Kasumi-1, KG-1a, MOLM-13, MV4-11, NB-4, Nomo-1, SKM-1, THP-1, U937) were obtained from the German Collection of Microorganisms and Cell Cultures (Braunschweig, Germany) and American Type Culture Collection (Manassas, VA, USA). Routine testing for mycoplasma contamination was performed every 3 months, and the authenticity was determined by the flow cytometry-based immunophenotyping according to the provider’s description.

### Antibody production and purification

The mouse anti-human CD276 8H8 mAb was generated, as previously described [5]. Chimerization (human immunoglobin G1/κ constant region) of the CD276 mAb 8H8 and control mAb MOPC21 was performed to generate 8H8_SDIE and its control MOPC_SDIE. Fc optimization (S293D/I332E modification) was introduced as described previously [27].

### Flow cytometry

Cells of interest were blocked with human (Merck KGaA, Darmstadt, Germany) or mouse (SouthernBiontech, Birmingham, AL, USA) IgG followed by incubation with mouse anti-human CD276 8H8 mAb, 8H8_SDIE, or the corresponding isotype controls (BD Pharmingen, San Diego, CA, USA), completed with goat anti-mouse-PE (DAKO, Glostrup, Denmark) or goat anti-human-PE (Jackson ImmunoResearch, West Grove, PA, USA). To detect AML blasts, patient PBMC was co-stained with CD33-BV510 (clone: WM53), CD34-BV421 (clone: 561) (both BioLegend, San Diego, CA, USA), and CD38-FITC (clone: HIT2) (BD Pharmingen). CD16-positive NK cells were identified using the fluorescent conjugates CD3-APC/Fire750 (clone: SK7), CD56-PECy7 (clone: HCD56), and CD16-APC (clone: 3G8) (BioLegend). For the detection of intracellular IFNγ, cells were cultured with BD GolgiStop and GolgiPlug (BD Biosciences, Heidelberg, Germany), stained for CD16 as described above, followed by fixation and permeabilization using the Fixation/Permeabilization Solution Kit according to the manufacturer’s instructions, and then stained with mouse anti-human IFNγ-PE (clone: B27) (BD Pharmingen). Target cell lysis was assessed by flow cytometry, as previously described [5]. Briefly, target cells were labeled with 2.5 µM CellTraceTM Violet cell proliferation dye (Thermo Fisher Scientific) before being seeded in co-cultures with PBMC from healthy donors, either with or without the antibodies (1 µg/mL each). Silicone beads (Merck KGaA) were used to ensure the measurement of equal test volumes. Dead cells were excluded from the analysis using 7-AAD (BioLegend) or LIVE/DEAD^TM^ Fixable Aqua (Thermo Fisher Scientific). Flow cytometric data were acquired using a FACS CANTO II or a FACS Fortessa (BD Biosciences) followed by data analysis using FlowJo v10 software (BD Biosciences). Specific fluorescence intensity (SFI) values were calculated by dividing the mean fluorescence intensity (MFI) of the measured antigen by the MFI of the corresponding isotype control. Surface positivity was defined as SFI ≥ 1.5. Additional information is provided in Supplemental Material.

### Analysis of NK cell activation, degranulation, and cytokine secretion

NK cell activation, degranulation, and cytokine secretion in PBMC from healthy donors were analyzed by co-culturing of 200,000 AML cells or patient-derived AML cells with 500,000 PBMC at an E:T ratio of 2.5:1 with indicated treatment (1 µg/mL). To determine NK cell degranulation, GolgiPlug and GolgiStop (BD Biosciences) were added to the co-cultures and cells were harvested after 4 hours (h), stained for CD107a-PE (clone: H4A3, BD Pharmingen) and analyzed by FACS. NK cell activation of cells harvested at 24 h and 72 h determined by measuring the expression of CD69-PE (clone: FN50, BD Pharmingen) and CD25-PE (clone: BC96, BioLegend) by flow cytometry. NK cells within PBMC were characterized as CD3^-^CD56^+^CD16^+^ subset. To investigate cytokine secretion by NK cells, supernatants from 24 h co-culture were collected and analyzed for the secretion of granzyme A, granzyme B, perforin, granulysin, TNF, IL-2, IFNγ, and IL-10 secretion using Legendplex (BioLegend) according to the manufacturer’s protocol. All experiments were performed as biological replicates from different donors.

### Analysis of NK cell cytotoxicity

Leukemic cell lysis by healthy donor PBMC with or without 8H8_SDIE/MOPC_SDIE (1 µg/mL) was analyzed using DELFIA Cell Cytotoxicity Assay (Perkin Elmer, Waltham, MA, USA) after 2 h of incubation according to established procedures [28]. The specific lysis rate was calculated using the formula:

100 × (experimental release−spontaneous release)/(maximum release−spontaneous release). Unless otherwise indicated, the lysis rates are the mean of technical triplicates with the standard error of the mean.

Long-term cytotoxicity analyses were performed using the Incucyte® S3 Live-Cell Analysis System (Essen Bioscience, Sartorius, Göttingen, Germany). Leukemic cells were labeled with Incucyte® Cytolight Rapid Red Dye (0.8 µM) for live cells and Incucyte® Cytotox Green Dye (25 nM) (both from Essen Bioscience Sartorius) to detect disruption of cell membrane integrity. Labeled target cells were then seeded in 96-well plates and co-cultured with PBMC from healthy donors (at an E:T ratio of 80:1) with or without the indicated mAbs (1 µg/mL). All experiments were performed as biological replicates from different donors and analyzed as technical triplicates. Images were taken every 4 h at 10× magnification. For quantification of dead cells, the dead target cell areas were normalized to the corresponding measurement at T = 0 h.

### Animal experiments

The mice used in this study were bred and maintained in a specific pathogen-free (SPF) environment at the University of Tübingen animal facility. In the in vivo toxicity model, NSG mice (NOD.Cg-*Prkd*^*scid*^ *Il2rg*^*tm1Wjl*^/SzJ, Charles River) (*n* = 4/group, males, 8-16 weeks old) were injected intravenously (i.v.) with 20 × 10^6^ human PBMC on day 1. Six hours later, the mice received additional i.v. injections of parental UCHT-1 Ab or 8H8_SDIE (20 µg each). After 24 h, cytokine levels in the mice sera were analyzed by Legendplex assay (BioLegend). In the in vivo AML tumor burden model, immunodeficient NSG mice (*n* = 5/group) were intravenously transplanted with luciferase-transduced U937 cells (1 × 10^6^ cells) (day 1), followed by injection of human PBMC (40 × 10^6^ cells, i.v.) 24 h later. At the same time, the animals were randomly treated with the 8H8_SDIE or its MOPC_SDIE control was introduced at 20 µg per mouse (day 2). To monitor AML tumor burden by bioimaging, mice were anesthetized with 5% isoflurane-oxygen mixture, 100 µl of D-luciferin (150 µg/ml) was injected intraperitoneally on day 4, 7, 11, and 14 and the luminescence was measured with an IVIS Lumina II imager (PerkinElmer). Animals that did not show engraftment by luminescence signal 24 h after AML injection were excluded from the analysis. Additional information is provided in Supplemental Material.

### Statistics

Unless otherwise noted, data are presented as mean ± standard error of the mean (SEM) of biological replicates or single values. All experiments, except animal models, were repeated in at least three different independent experiments. Data were tested for distribution using the Shapiro-Wilk test. Statistically significant differences of normally distributed data were calculated using Student’s t-test, one-way ANOVA, and for non-parametric data, Kruskal-Wallis, Friedman, Mann-Whitney, or log-rank test. Dunn’s test was used for correction of multiple comparisons. Data were analyzed using GraphPad Prism 9.4.1, and outliers were defined by the ROUT method (Q = 1%). Statistical tests were considered significant if the p-value was less than 0.05 (*p < 0.05, **p < 0.01, ***p < 0.001).

## Results

### CD276 surface expression on AML cell lines and primary leukemic cells as detected by the CD276 binder 8H8

**Fig. 1:**
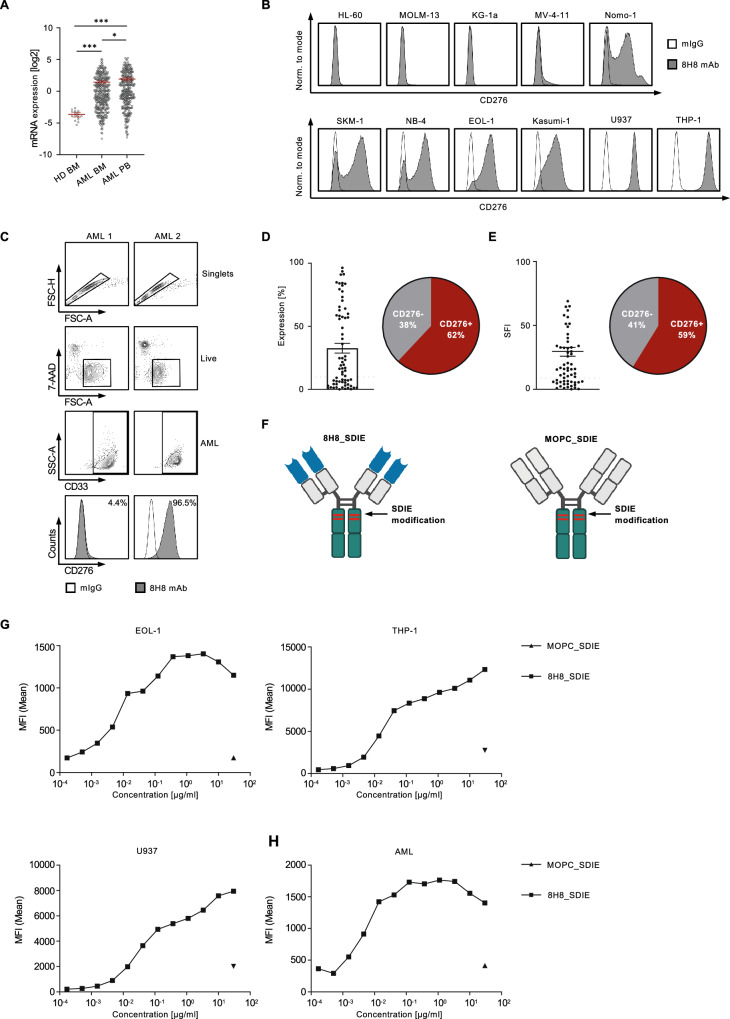
Characterization of CD276 expression in AML cell lines and patient-derived leukemia cells. **A** CD276 mRNA expression in AML bone marrow (BM) (*n* = 350) and AML peripheral blood (PB) (*n* = 318) and healthy BM (*n* = 16) samples were analyzed using the public data sets of the BeatAML program [29]. **B** Flow cytometric analysis of surface CD276 expression on the indicated AML cell lines using chimeric mouse anti-human 8H8 mAb against CD276 and corresponding isotype control. Example histograms of one representative experiment out of three with similar results are shown. **C** The gating strategy for two representative patient-derived AML samples is outlined as follows: singlets, viable (7-AAD^-^), leukemic blast marker (CD33^+^), and the percentage of CD276 expression. **D** CD276 expression on AML patient blasts (*n* = 68) is shown as percentage of CD276^+^ AML blasts and **E** as SFI levels. Expression levels above SFI of 1.5 and 10% for positive cells were considered as positive expression (indicated by the dotted line and shown by the corresponding pie chart). **F** Schematic representation of the generated CD276 antibody with a modified Fc part optimized for increased affinity to CD16 (8H8_SDIE) and the corresponding control mAb (MOPC_SDIE). **G** Titration of the 8H8_SDIE mAb indicated AML cell lines and **H** on AML patient sample analyzed by flow cytometry. MOPC_SDIE was used as an isotype control. The example data show the MFI values of one representative experiment out of a total of three with comparable results.

First, we examined data sets from the BeatAML project [29] to analyze the mRNA expression levels of CD276 in AML bone marrow (BM) and peripheral blood (PB) samples compared to healthy donor BM samples. The analysis included data sets of 16 healthy donor BM, 350 AML BM, and 318 AML PB samples. Significantly higher CD276 mRNA expression was detected in all data sets of malignant cells compared to normal BM samples (Fig. 1A). Next, we evaluated the specific binding of the murine CD276 hybridoma-derived antibody clone 8H8 [5] to assess the surface expression of CD276 on various leukemia cell lines representing a broad spectrum of myeloid malignancies, such as EOL-1, HL-60, Kasumi-1, KG-1a, MOLM-13, MV-4-11, NB-4, Nomo-1, SKM-1, THP-1 and U937. Flow cytometric analysis revealed substantial CD276 expression on NOMO-1, SKM-1, NB-4, EOL-1, Kasumi-1, U937, and THP-1 cells, whereas the HL-60, MOLM-13, KG-1a, and MV-4-11 cells lacked CD276 expression (Fig. 1B). Next, the CD276 expression was analyzed on leukemic blasts from 68 AML patients. AML patient characteristics are shown in Table 1. The gating strategy and surface staining are shown in Fig. 1C. CD276 expression ≥ 10% was considered positive and was detected in 62% of patient samples (42 of 68) (Fig. 1D). By defining AML samples with SFI ≥ 1.5 as CD276 positive, relevant CD276 surface expression was detected in 59% (40 out of 68) of all AML cases (Fig. 1E).

**Table 1:** AML patient characteristics.

	Number of patients (*n* = 68)
Age at diagnosis [years]	61.7 (range 26.0–89.0)
Sex [%]	
Female	48.5 (33)
Male	51.5 (35)
Primary/Secondary AML [%]	
Primary	77.9 (53)
Secondary	14.7 (10)
Unknown	7.4 (5)
FAB classification [%]	
M0	4.4 (3)
M1	25.0 (17)
M2	22.1 (15)
M3	0 (0)
M4	19.1 (13)
M5	25.0 (17)
Unknown	4.4 (3)
Blood count at diagnosis	
PBB [%]	74.2 (range 2.0–100.0)
WBC [G/L]	123.6 (range 2.2–448.3)
Hb [g/dL]	8.5 (range 3.8–13.2)
Plt [G/L]	69.2 (range 6.0–433.0)

*FAB* French-American-British, *PBB* Peripheral Blood Blast, *WBC* White blood cells, *Hb* Hemoglobin, *Plt* Platelets.

Next, we functionally characterized a humanized CD276 mAb clone 8H8 termed 8H8_SDIE containing the S239D/I332E modification (8H8_SDIE) to increase the affinity to the Fc receptor CD16 on NK cells (Fig. 1F, the left panel) [30]. An Fc-optimized control protein with non-relevant target specificity, termed MOPC_SDIE, was used as control (Fig. 1F, the right panel). Binding of (8H8_SDIE) to AML cells was determined by dose titration experiments using three AML cell lines (EOL-1, THP-1, U937) (Fig. 1G) and a primary AML patient sample (Fig. 1H). In all cases, a dose of approximately 1 µg/mL was sufficient to achieve saturated mAb binding.

### Induction of NK cell reactivity against CD276^+^ AML cell lines

**Fig. 2:**
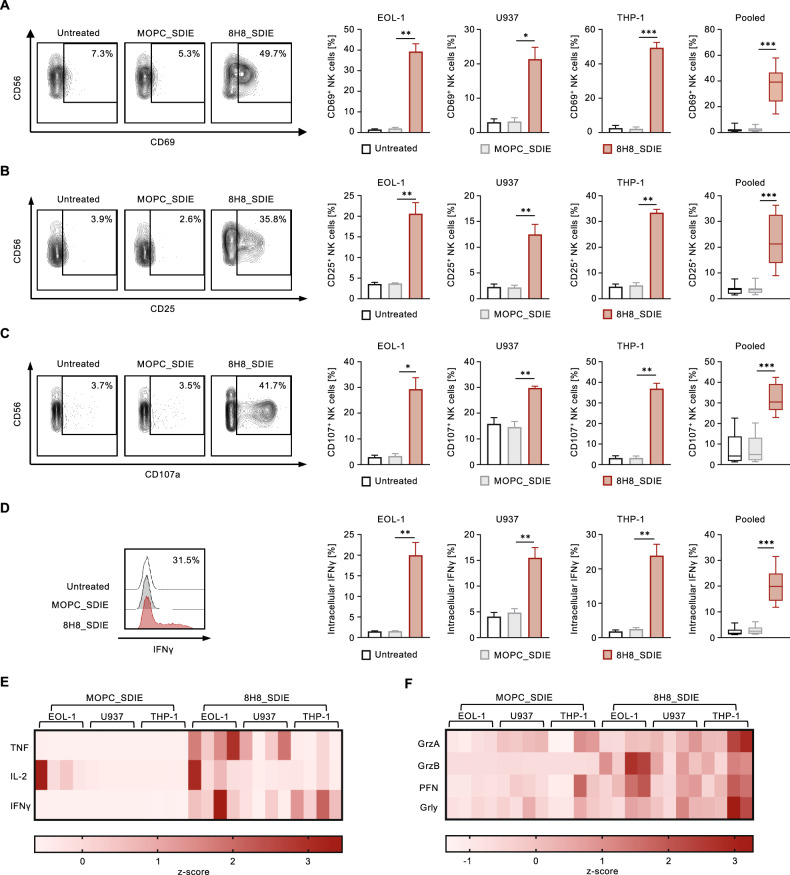
Induction of NK cell reactivity by Fc-optimized CD276 antibody against AML cell lines. PBMC from healthy donors were co-cultured (E:T 2.5:1) in the presence or absence of 8H8_SDIE antibody or the MOPC_SDIE control (both 1 µg/mL). **A** NK cell activation was analyzed by CD69 expression after 24 h. The left panels show representative flow cytometric results of THP-1 cells, the right panels show separate and pooled data of the indicated leukemic cell lines incubated with PBMC (*n* = 4). **B** NK cell activation was determined by CD25 expression after 72 h. The left panels show representative flow cytometric results of THP-1 cells, the right panels show individual and pooled data of the indicated leukemic cell lines incubated with PBMC (*n* = 4). **C** NK cell degranulation was analyzed by CD107a expression after 4 h. The left panels show representative flow cytometric results of THP-1 cells, the right panels show individual and pooled data of the indicated leukemic cell lines with PBMC (*n* = 4). **D** Intracellular IFNγ expression of NK cells within PBMC was characterized by CD3^-^CD56^+^CD16^+^ counterstaining and determined by flow cytometry after 4 h. The left panel shows exemplary data from THP-1 cells, the right panels show individual and pooled data of the indicated leukemic cell lines incubated with PBMC (*n* = 4). **E**, **F** Supernatants of the respective co-cultures were analyzed after 24 h for the release of the immunoregulatory molecules TNF, IL-2, IFNγ and for the effector molecules granzyme A (GrzA), granzyme B (GrzB), perforin (PFN) and granulysin (Grly) by Legendplex assay. The heatmap diagrams show individual results for the leukemic cell lines indicated and different PBMC (*n* = 4).

Next, we investigated the potential of 8H8_SDIE to induce NK cell-mediated anti-leukemia responses. To this end, PBMC obtained from healthy donors, containing NK cells as effector cells, were co-cultured with different AML cell lines in the absence or presence of 8H8_SDIE or its isotype control MOPC_SDIE. Flow cytometric analysis of NK cells within PBMC showed significantly increased CD69 expression after 24 h of treatment with 8H8_SDIE, indicating enhanced NK cell activation with all AML cell lines tested. The MOPC_SDIE control had no significant effect (Fig. 2A). After 72 h of incubation, treatment with 8H8_SDIE resulted in a significant increase in CD25 expression, and again no effect was observed with the isotype control (Fig. 2B). The enhanced NK cell activation induced by 8H8_SDIE was reflected by a pronounced degranulation as analyzed by CD107a expression in co-cultures with PBMC with all AML cell lines tested, while MOPC_SDIE had no relevant effect (Fig. 2C).

IFNγ is a cytokine that not only exerts direct anti-tumor effects, but also plays a crucial role in subsequent adaptive immune responses [31]. Intracellular flow cytometry analysis revealed a significant increase in IFNγ production in NK cells after 8H8_SDIE mAb treatment (Fig. 2D). The increased intracellular IFNγ levels were reflected by release of cytokines in the culture supernatants. IFNγ, TNF, and IL-2 were released at higher levels after 8H8_SDIE treatment (Fig. 2E). In addition, we observed increased levels of cytotoxic molecules such as granzyme A, granzyme B, perforin, and granulysin after 8H8_SDIE treatment compared to control treatment (Fig. 2F).

### Induction of NK cell cytotoxicity against CD276^+^ AML cell lines

**Fig. 3:**
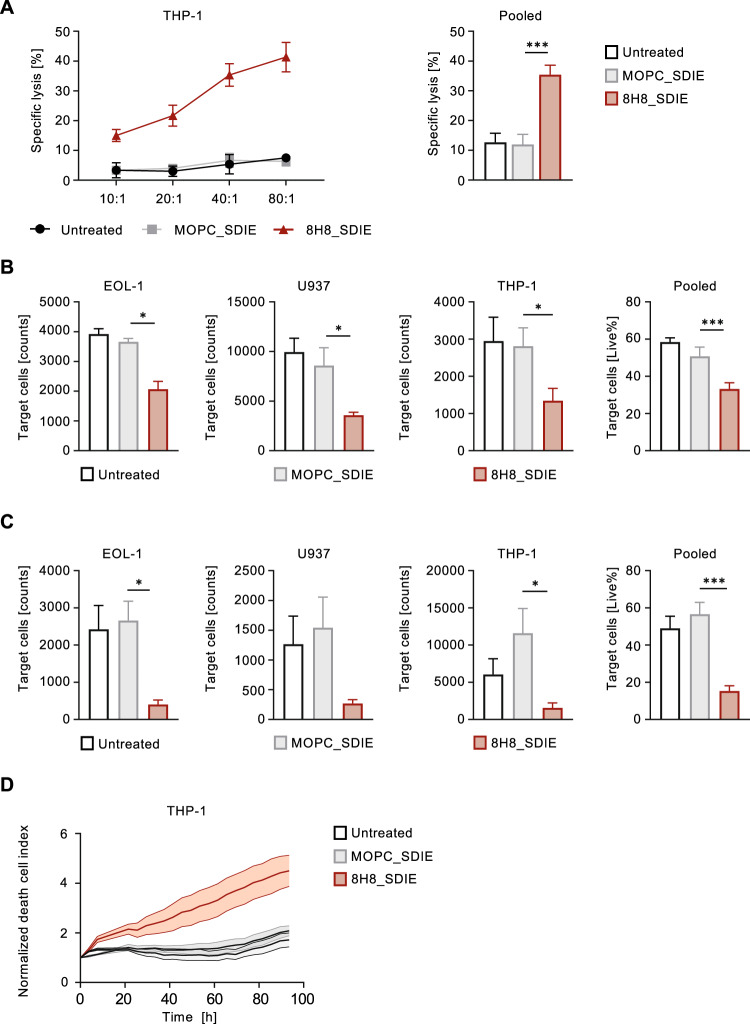
Induction of targeted cell lysis in AML by 8H8_SDIE. PBMC from healthy donors were co-cultured with the indicated leukemic cells in the presence or absence of 8H8_SDIE mAb or MOPC_SDIE control (both 1 µg/mL). **A** Targeted lysis of AML cells was determined by Europium-based cytotoxicity assays after 2 h of incubation. The left panel shows example data with THP-1 cells and a PBMC donor at different E:T ratios. The right panel shows pooled data from EOL-1, U937 and THP-1 cell lines with different PBMC donors (*n* = 6-9) (E:T 80:1). **B** Lysis of AML cell lines with different PBMC donors (*n* = 3-4) was analyzed by flow cytometry after 24 h (E:T 20:1). **C** Lysis of the different AML cell lines with PBMC from healthy donors (*n* = 3-4) was analyzed by flow cytometry after 72 h (E:T 20:1). **D** Survival of AML cell lines was determined using a live cell imaging system. THP-1 cells were cultured with PBMC from healthy donors (*n* = 4) (E:T 80:1) for 96 h. Dead target cell areas were normalized to the initial target cell area’s initial measurement at T = 0 h.

Next, we investigated whether the increased NK cell reactivity induced by 8H8_SDIE results in increased target cell lysis. PBMC from healthy donors were co-cultured with three AML cell lines in the presence or absence of 8H8_SDIE mAb. Europium-based short-term cytotoxicity assays showed that 8H8_SDIE significantly increased target cell lysis after 2 h with all AML cell lines tested (Fig. 3A). Similarly, long-term 24 and 72 h flow cytometry-based lysis assays documented the potency of 8H8_SDIE to induce lysis of leukemic cells (Fig. 3B, C), and the ability of 8H8_SDIE to induce target cell lysis was further validated over a 96 h period using live cell imaging (Fig. 3D).

### Induction of NK cell reactivity and cytotoxicity against CD276^+^ primary AML cells

**Fig. 4:**
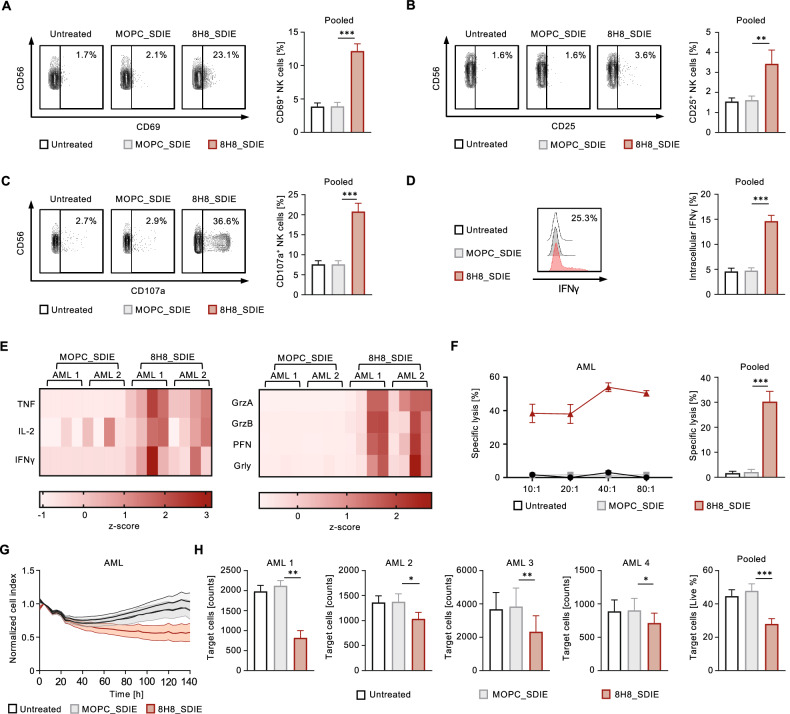
Induction of NK cell reactivity and cytotoxicity by Fc-optimized CD276 antibody against primary AML cells. PBMC from healthy donors were co-cultured with primary AML cells in the presence or absence of 8H8_SDIE or MOPC_SDIE control (both 1 µg/mL) (E:T 2.5:1). **A**–**D** The left panels show representative flow cytometry results obtained from an AML patient sample and a PBMC donor, the right panel shows combined data from AML patients (*n* = 6) and PBMC from healthy donors (*n* = 4). **A** NK cell activation was analyzed based on the CD69 expression after 24 h. **B** NK cell activation was determined by the CD25 expression after 72 h. **C** NK cell degranulation was determined by the CD107a expression after 4 h. **D** Intracellular IFNγ expression in NK cells within PBMC, characterized by CD3^-^CD56^+^CD16^+^ counterstaining, was determined by FACS after 4 h. **E** Culture supernatants were analyzed after 4 h for release of immunoregulatory molecules TNF, IL-2, IFNγ and for the effector molecules granzyme A (GrzA), granzyme B (GrzB), perforin (PFN), granulysin (Grly) by Legendplex assays. The heatmap plots show results for AML patients (*n* = 2) with PBMC from healthy donors (*n* = 4). **F** Targeted lysis of primary AML cells was determined by Europium cytotoxicity assays after 2 h of incubation. In the left panel exemplary data obtained from a healthy PBMC donor and a primary AML sample at different E:T ratios is shown. In the right panel, pooled data for primary AML cells (*n* = 3) were obtained with PBMC from healthy donors (*n* = 3) (E:T 80:1). **G** Survival of primary AML cells was determined using a live cell imaging system. The primary AML sample was cultured with PBMC from 4 healthy donors (E:T 80:1) for 140 h. The live target cell areas were normalized to the respective initial target cell area’s initial measurement at T = 4 h. **H** Lysis of primary AML cells with PBMC from healthy donors (*n* = 4) was analyzed by flow cytometry after 72 h (E:T 20:1). In the left panel, separate data for each AML patient with PBMC from healthy donors (*n* = 4), in the right panel pooled data for all AML patients (*n* = 4) (E:T 20:1) are shown.

Next, we evaluated the efficacy of 8H8_SDIE to induce NK cell reactivity and cytotoxicity against primary AML cells. When healthy donor PBMC were co-cultured with patient AML cells, the presence of 8H8_SDIE resulted in significantly increased activation and degranulation as measured by CD69 as well as CD25 expression and degranulation (CD107a) of NK cells, respectively (Fig. 4A–C). 8H8_SDIE also robustly induced intracellular IFNγ within NK cells (Fig. 4D) and release of the immunomodulatory cytokines and cytotoxic molecules as determined by Legendplex assays (Fig. 4E). Results obtained from short-term cytotoxicity assays showed that exposure to our 8H8_SDIE construct profoundly enhanced cell lysis in all patient-derived AML cells tested (Fig. 4F). Long-term efficacy of target-specific lysis efficacy was confirmed in 72 h and 140 h assays (Fig. 4G, H).

### In vivo evaluation of the 8H8_SDIE construct

**Fig. 5:**
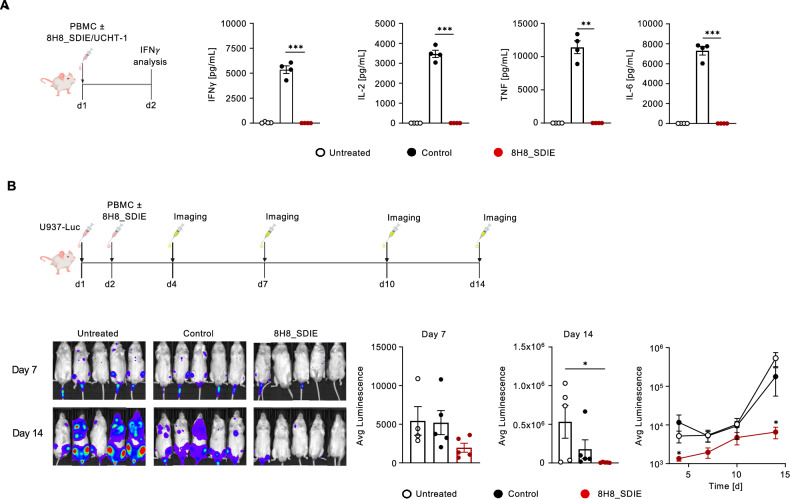
Determination of toxicity and efficacy of the 8H8_SDIE mAb in vivo. **A** Immunocompromised NSG mice (*n* = 4/group) were injected with human PBMC (20 × 10^6^ cells, i.v.) and treated with an CD3 antibody (clone UCHT1, 20 µg, i.v.) as control or 8H8_SDIE (20 µg, i.v.). Blood was collected 24 h after injection and serum levels of IFNγ, IL-2, TNF, IL-6 were measured by Legendplex. **B** Luciferase-expressing U937 cells (1 × 10^6^) were injected intravenously into NSG mice (*n* = 5/group) followed by PBMC injection (40 × 10^6^, i.v.) and 8H8_SDIE (20 µg, i.v.) on the next day. AML tumor burden was monitored over time by bioluminescence imaging. The left panel shows exemplary bioluminescence images of mice from the indicated treatment groups on days 7 and 14. The middle panel shows the quantitative analysis of AML tumor burden measured by luminescence for all treatment groups on days 7 and 14. The right panel shows the AML tumor burden measured twice a week over the 2-week period.

Potential in vivo cytotoxicity of 8H8_SDIE, which could be mediated by off-target immune cell activation in the absence of tumor targets, could lead to severe side effects in patients. To rule out unwanted off-target activity of our new construct, we injected immunocompromised NSG mice reconstituted with human PBMC from healthy donors with 8H8_SDIE and the agonistic CD3 antibody UCHT1, which induces potent T cell activation and thereby massive cytokine release. Analysis of serum levels of IFNγ, IL-2, TNF and IL-6 showed no off-target effects of 8H8_SDIE, whereas the positive control potently induced cytokine release (Fig. 5A). We then investigated the anti-leukemic potential of 8H8_SDIE by analyzing the AML tumor burden after engraftment of luciferase-expressing U937 cells in NSG mice followed by the transfer of human PBMC in the presence or absence of the constructs. Monitoring of AML tumor burden by bioluminescence imaging over a period of 2 weeks demonstrated that 8H8_SDIE inhibited leukemia development. In contrast, the control group of mice showed pronounced leukemia outgrowth (Fig. 5B).

## Discussion

Immunotherapy has emerged as a promising treatment for many disease entities, but has not yet been established in AML. Approaches such as CAR-T cell therapy, bispecific T cell engagers (BiTEs), checkpoint inhibitors, and immunomodulatory agents have demonstrated efficacy in both preclinical and clinical settings. However, an established treatment to achieve cure for AML is still lacking. Therefore, further research is needed to refine and improve immunotherapeutic techniques to determine the most effective treatment options for AML patients [32].

In this study, we report the preclinical evaluation of an Fc-optimized mAb termed 8H8_SDIE, targeting CD276 for AML treatment. We documented CD276 expression in various AML cell lines and primary AML patient samples. 8H8_SDIE showed excellent binding to AML cells and exhibited potent anti-tumor efficacy against AML cell lines and patient cells in vitro. In addition, in vivo experiments in immunocompromised NSG mice engrafted with human PBMC showed no off-target immune activation and pronounced anti-leukemia activity, underscoring the potential of 8H8_SDIE as therapeutic agent for AML treatment.

The pivotal role of NK cells in mediating ADCC upon treatment with therapeutic mAbs has been clearly established. Current research is focused on improving the efficacy with an emphasis on enhancing ADCC. A key strategy is to optimize the Fc part by enhancing its interaction with CD16. This offers a promising avenue for refining anti-tumor antibody therapies to improve clinical outcomes. The SDIE modification of the Fc part represents a significant advance in the enhancement of NK cell-mediated ADCC. Several Fc-optimized mAbs containing the SDIE modification are successfully undergoing clinical evaluation. Examples of such mAbs include Margetuximab (Her2; NCT01828021), MEN1112 (CD157; NCT02353143), FLYSYN (anti-FLT3; NCT02789254) [14] and BI 836858 (CD33; NCT02240706, NCT03013998), and Tafasitamab-cxix (CD19; NCT02399085) has recently received FDA approval.

CD276 has attracted considerable attention as target antigen for immunotherapeutic approaches. It has been implicated as inhibitory immune checkpoint, although currently available data regarding its precise functional role are somewhat conflicting [33–35]. Initially, CD276 was described as a costimulatory molecule that enhances the proliferation of CD4^+^ and CD8^+^ T cells in the presence of an CD3 antibody [36]. Consistently, the introduction of CD276 into mice with colorectal cancer was shown to inhibit tumor growth and reduce secondary metastasis [37]. In contrast, a number of studies have shown that CD276 inhibits anti-tumor immunity, including suppression of CD4^+^ and CD8^+^ T cell activation and proliferation, and reduction of IL-2 and IFNγ production. In addition, a recent study indicates that CD276 facilitates evasion of immune surveillance at various stages of cancer, including initiation, development, and metastasis of head and neck squamous cell carcinoma. This suggests that the immunosuppressive environment influenced by CD276 helps cancer cells evade destruction by immune cells [31]. Because of its putative immunosuppressive role and its broad but relatively tumor-specific expression CD276 is an attractive target for antibody-based immunotherapy [38, 39]. In light of these findings, several immunotherapeutic strategies targeting CD276 are currently under clinical investigation. These include, but are not limited to, antibody-drug conjugates (e.g., MGC018: NCT037219596; DS7300a: NCT04145622), radiolabeled mAbs (e.g., ^131^I-8H9: NCT03275402, NCT04022213; ^177^Lu-DPTA Omburtamab: NCT04315246, NCT04167618), Fc-optimized mAbs (such as MGA271, Enoblituzumab: NCT02923180, NCT02475213, NCT04634825; DS-5573a: NCT02192567, clinical trial discontinued), and bispecific antibodies, such as the B7-H3xCD3 for the treatment of gastrointestinal cancers [30, 40]. Our study adds the Fc-optimized mAb 8H8_SDIE to this panel of immunotherapeutic agents and documents its significant potential for effective induction of NK cell-mediated ADCC.

When considering the potential toxicity and side effects of 8H8_SDIE, it is important to note that CD276 is not exclusively expressed on tumor cells [36]. Basal expression of CD276 has been reported on several healthy cell types, including liver, endothelial cells, amniotic fluid stem cells, quiescent fibroblasts and osteoblasts [41]. When CD276 has been used as a therapeutic target in preclinical studies, including the use of CD276-directed CAR-T cells, significant anti-tumor effects have been observed [42–46]. In line, clinical trials investigating immunotherapies targeting CD276, such as the bispecific antibody (bsAb) MGD009, have shown minimal toxicity. This may be attributed to the aggregation of numerous single chains, leading to off-target T cell activation and cytokine release. Additionally, there is a potential for “on-target, off-tumor” toxicity with bsAbs, given the limited expression of CD276 on endothelial cells [40].

Our mAb 8H8_SDIE demonstrated robust anti-leukemic efficacy during in vitro evaluations using various AML cell lines and patient leukemia cells, which was confirmed by in vivo experiments in xenograft models. The results presented in this study thus highlight the potential of our Fc-optimized mAb targeting CD276 for the treatment of AML.

### Supplementary information

Supplemental Material

## Data Availability

The data sets supporting the conclusions of this article are included in the article. Additional data could be provided by the corresponding author upon reasonable request.
