# Supplementary material for: Induction of NK cell reactivity against acute myeloid leukemia by Fc-optimized CD276 (B7-H3) antibody
Source: Blood Cancer J. 2024 Apr 18;14(1):67. doi: 10.1038/s41408-024-01050-6 (PMC11026476; doi:10.1038/s41408-024-01050-6)
Supplement: Supplementary file 1 — Supplemental Material [file 41408_2024_1050_MOESM1_ESM.pdf]

Supplemental Material

Key resources table

**Reagent or Resource**	**Vendor**	**Cat. number**
**Antibodies**		
human IgG	Merck KGaA	I4506
mouse IgG-UNLB	SouthernBiontech	0107-01
mouse IgG1	BD Pharmingen	557273
goat anti-mouse IgG, F(ab')$_2$	DAKO	R0480
goat anti-Human IgG, F(ab')$_2$	Jackson ImmunoResearch	109-116-097
mouse anti-human CD33 BV510	BioLegend	303422
mouse anti-human CD34 BV421	BioLegend	343610
mouse anti-human CD38 FITC	BD Pharmingen	555459
mouse anti-human CD3 APC/Fire750	BioLegend	344840
mouse anti-human CD56 PECy7	BioLegend	318318
mouse anti-human CD16 APC	BioLegend	302011
mouse anti-human IFNγ PE	BD Pharmingen	554701
7-AAD Viability Staining Solution	BioLegend	420404
LIVE/DEAD™ Fixable Aqua Dead Cell Stain Kit	Thermo Fisher Scientific	L34966
mouse anti-human CD107a PE	BD Pharmingen	555801
mouse anti-human CD69 PE	BD Pharmingen	555531
mouse anti-human CD25 PE	BioLegend	302606
mouse anti-human CD3 Ultra-LEAF	BioLegend	317326
**Chemicals**		
BD GolgiPlug™	BD Biosciences	555029
BD GolgiStop™	BD Biosciences	554724
BD Cytofix/Cytoperm™	BD Biosciences	554722
CellTrace™ Violet Cell Proliferation Kit	Thermo Fisher Scientific	C34557
Latex beads, polystyrene	Merck KGaA	LB30
LEGENDplex™ Human CD8/NK Panel (13-plex)	BioLegend	740267
DELFIA BATDA Reagent	Perkin Elmer	C136-100
DELFIA Europium Solution	Perkin Elmer	C135-100
Incucyte® CytoLight Rapid Red	Bioscience Sartorius	4706
Incucyte® Cytotox Green Dye	Bioscience Sartorius	4633
D-Luciferin sodium salt	abcam	ab145164
**Experimental model strains**		
NOD.Cg-*Prkd*^{scid} *Il2rg*^{tm1Wjl}/SzJ	Charles River	Strain: 005557

In vivo study design

The *in vivo* experiment's sample size was determined through careful power calculations based on prior published studies. Whenever possible, littermate controls were used. Both control and experimental mice were matched for age (8-16 weeks old) and sex. All experiment-generated data were thoroughly analyzed, and experimental results were consistently replicated, with the number of replicates clearly indicated in the figure legends.

Rigorous blinding procedures were implemented to minimize bias in animal experiments. In feasible blind experiments, steps were taken to ensure that the experimenter was unaware of the treatment of the animals during the data collection phase. However, it is important to note that in certain cases blinding was not feasible due to limitations in the experimental design or the nature of the measurements involved, as illustrated by the different groups of mice treated with 8H8_SDIE or its MOPC_SDIE control.
